# Adaptive microevolution in CC17 *Streptococcus agalactiae* from a mother-neonate infection

**DOI:** 10.3389/fcimb.2026.1926326

**Published:** 2026-09-14

**Authors:** Jingxian Liu, Yixin Zhu, Jiajia Yu, Ying Liu, Feng Chen, Yingxia Zheng

**Affiliations:** 1 Department of Clinical Laboratory, Xin Hua Hospital, Shanghai Jiao Tong University School of Medicine, Shanghai, China; 2 Institute of Artificial Intelligence Medicine, Shanghai Academy of Experimental Medicine, Shanghai, China; 3 Faculty of Medical Laboratory Science, College of Health Science and Technology, Shanghai Jiao Tong University School of Medicine, Shanghai, China

**Keywords:** adaptive microevolution, infection, *Streptococcus agalactiae*, suppurative arthritis, virulence

## Abstract

**Background:**

The hypervirulent CC17 GBS is a major cause of neonatal invasive disease. Although this lineage can acquire mutations during host adaptation, the functional significance of such microvariations for bacterial pathogenesis remains unclear.

**Methods:**

The GBS strains were isolated from a mother-neonate pair, with clinical data obtained from electronic medical record. MALDI-TOF MS was used for species identification. Antimicrobial susceptibility testing was performed using K-B method and interpreted by CLSI criteria. Serotyping and MLST were used for molecular epidemiological analysis. Growth curve, hemolysin activity, serum inhibition and hyaluronic acid survival assays were performed to evaluate phenotypic differences between strains. Cell invasion assays and *Galleria mellonella* larval infection model were used to assess the invasiveness and *in vivo* virulence of GBS strains. Whole-genome sequencing was applied to compare the genetic backgrounds of the strains. Transcriptome sequencing and rtPCR were conducted to identify and validate DEGs between isolates.

**Results:**

A total of 5 GBS strains were collected and identified, and all were susceptible to penicillin, but resistant to clindamycin and erythromycin. The strains could be classified into ST17 and ST2247. The two types of strains showed no significant differences in growth curve, hemolysis activity, or serum inhibition. In hyaluronic acid solution, the ST2247 strain exhibited higher viability compared to the ST17 (*P<0.05*). The invasive ability of the ST2247 strain against hUVEC, HTR-8/svneo, or hCMEC/D3 was weaker than that of the ST17 (*P<0.05*). However, ST2247-infected *Galleria mellonella* larval had a significantly lower vitality index and higher mortality rate than ST17-infected ones (*P<0.05*). ST17 and ST2247 exhibited consistent carriage of virulence genes, resistance genes and prophage elements, while only 5 SNPs and 3 INDELs were identified between them, with one single-nucleotide insertion (C>CA) locates 75 bp upstream of the *ptsG* start codon. Transcriptome sequencing and rt-PCR showed that the expression level of the *PTSIIBC* gene in ST2247 strain was much higher than that in ST17 strain (*P<0.05*).

**Conclusion:**

ST17 GBS can experience microevolution within maternal and infant hosts, evolving through minor genomic mutations into new strains. Variations in *PTSIIBC* gene may associated to the divergence in adaptability and virulence of CC17 GBS strains.

## 1 Introduction

*Streptococcus agalactiae* (also known as group B streptococcus, GBS) is one of the most common pathogens causing neonatal infections. Widely recognized as a hypervirulent clone, clonal complex (CC)17 is the predominant lineage causing neonatal sepsis and meningitis globally (Hsu et al., 2023; Choi et al., 2024). Currently, intrapartum antibiotic prophylaxis (IAP) is the core measure for preventing infant GBS infection, and has substantially reduced the risk of early-onset GBS disease (EOD) (Panneflek et al., 2024). However, epidemiological data indicate that in the context of IAP prevention and control, the prevalence of well-documented hypervirulent CC17 strain is continuously expanding, and the molecular and evolutionary mechanisms underlying this phenomenon remain unclear (Khan et al., 2024).

During colonization, transmission and infection within the host, bacteria continuously respond to external selective pressures, leading to genomic variations and phenotypic remodeling, which is known as within-host microevolution (Tonkin-Hill et al., 2025; Wang et al., 2026). Host immunity, antibiotic exposure and tissue microenvironmental differences drive bacterial adaptive evolution, which modulates their virulence, antibiotic resistance and immune evasion, and further influence infection progression and clinical outcomes (Keogh et al., 2022). Several research teams have performed whole genome sequencing tracking of GBS isolates from pregnant women receiving IAP. Their results confirmed the occurrence of in-host genomic mutations in GBS (Pell et al., 2026). Nevertheless, current studies merely describe genomic variation profiles and fail to clarify the relationships between genetic mutations and virulence phenotypes, as well as the underlying regulatory mechanisms.

This study identified a unique mother-neonate pair, from which GBS strains of two different sequence types (STs) were isolated from multiple clinical specimens, providing an ideal model for microevolutionary study. By integrating phenotypic experiments, virulence characterization, WGS and transcriptomic analysis, this study focuses on the CC17 lineage to characterize the microevolutionary features of the strains during cross-host transmission, and to elucidate the molecular mechanisms underlying the pathogenicity evolution mediated by selective pressure. This work provides experimental evidence to better understand mother-to-child transmission patterns of GBS and to investigate the potential drivers for the predominance of hypervirulent CC17 strains.

## 2 Materials and methods

### 2.1 Isolates collection and identification

On March 24 2020, a neonate was admitted to Xinhua Hospital for decreased movement of the right lower limb, his clinical information was collected through the electronic medical record system. *S. agalactiae* was isolated from his joint fluid, bloodstream, throat swab and his mother’s breast milk and vaginal swab. The strains were identified by matrix-assisted laser desorption/ionization-time of flight mass spectrometry (MALDI-TOF MS; Microflex™ LT, Bruker Daltonik, Germany). For each positive specimen, at least five isolated colonies were randomly picked from the primary culture plates. Each colony was independently subcultured and stored at −80 °C in glycerin broth. Isolates were then recovered and cultured on 5% sheep blood plates for 24 h at 37 °C in a 5% CO2 atmosphere.

### 2.2 Antimicrobial susceptibility testing

According to the protocols provided by the Clinical and Laboratory Standards Institute (CLSI, 2020), the antimicrobial susceptibility of the isolates to penicillin, ceftriaxone, erythromycin, clindamycin, tetracycline, ciprofloxacin, linezolid and vancomycin was determined by disk diffusion in sheep blood Mueller-Hinton agar plates (Oxoid, UK). The D test was performed to determine inducible clindamycin resistance. ATCC12386 strain were used for quality control.

### 2.3 Serotyping

The serotypes (Ia, Ib, II–VII) based on capsular polysaccharide (CPS) were distinguished using previously developed multiplex polymerase chain reaction (PCR) methods (Poyart et al., 2007).

### 2.4 Multilocus sequence typing

MLST was performed by sequencing seven housekeeping genes *adhP*, *pheS*, *atr*, *glnA*, *sdhA*, *glcK*, and *tkt*, as previously described (Jones et al., 2003). The ST was determined using the *S. agalactiae* MLST database. New alleles or ST profiles were submitted and assigned to the *S. agalactiae*. For each specimen, two independently preserved colonies were recovered and analyzed in parallel by MLST.

### 2.5 Determination of growth curve and inoculum quantification

The growth kinetics of GBS isolates were evaluated via turbidimetric assay. Briefly, a single colony of GBS was inoculated into 5 mL of Brain Heart infusion (BHI) and incubated overnight at 37 °C with 5% CO_2_ to prepare a seed culture. The McFarland units (McF) was measured hourly using a calibrated McFarland densitometer (Biomérieux, France) for the first 12 hours to capture the exponential growth phase, followed by a final measurement at 24 hours post-inoculation to assess late stationary phase (Mahesh et al., 2025). The growth curve was constructed by plotting the mean McF values against time, with standard deviations represented as error bars.

The strains were harvested at the mid-log phase by centrifugation. After washing and resuspension in PBS, the bacterial suspension was adjusted to 1.0 McF, and its corresponding viable count (CFU/mL) was verified by serial dilution and agar plating.

### 2.6 Hemolysin activity determination

The GBS strains were incubated in BHI at 37 °C overnight in a shaker. Supernatants were collected from bacterial cultures grown for 48 h with optical density values normalized at 600 nm. Hemolytic activities were determined by incubating supernatant of selected strains with 3% sterile defibrillation rabbit blood in phosphate-buffered saline (PBS) for 1 h at 37 °C. Ultimately, the red blood cells deposited were discarded after centrifugation, and hemolysis was semi-quantified by measuring the optical density of the supernatant at 600 nm as previously described (Wang et al., 2023). 10% SDS was used as a positive control. The assay was performed in triplicate.

### 2.7 Serum killing assay

GBS strains were cultured in BHI to the postexponential phase. Approximately 10^6^ CFU of the bacteria were harvested and resuspended in fresh heparinized human serum, followed by incubation at 37 °C with constant rotation for 1, 2, and 3 hours, respectively. After incubation, serially diluted samples were spread onto nutrient agar plates to enumerate viable bacteria as previously described (Qiu et al., 2023). The entire assay was independently repeated three times to ensure result reliability.

### 2.8 Survival assay

GBS strains were cultured in BHI to the logarithmic growth phase, centrifuged to discard the supernatant, and the bacterial cells were collected and washed twice with 1×PBS. The bacterial pellet was resuspended in PBS to prepare a suspension with a concentration of approximately 10^7^ CFU/ml. This suspension was added to glucose (GLU) solution, hyaluronic acid (HA) + GLU solution, and HA solution at a ratio of 1:10, resulting in a final bacterial concentration of 10^6^ CFU/ml, a final HA concentration of 3mg/ml and GLU 1mg/ml. The mixtures were incubated overnight at 35°C in a 5% CO_2_ environment. Then the solutions were diluted and spread on BHI agar, which were then incubated overnight at 35°C in a 5% CO_2_ environment before counting the number of colonies on the plates.

### 2.9 Cytotoxicity assay

Following the manufacturer’s protocol of high-sensitivity rapid CCK-8 detection kit (Servicebio, Wuhan, China), cells were seeded in a 96-well cell culture plate and allowed to reach over 90% confluency. After washing with PBS, bacterial suspension was introduced at a final concentration of 10^6^ CFU/mL. Three control groups were included in this assay, namely the medium blank control (A_blank_), the bacteria-only control (A_bacteria-only_), and the uninfected cell-only control (A_cell-control_). Following a 2-hour incubation period, 10 µL of hypersensitive CCK-8 reagent was added to each well. The plate was then incubated for an additional 0.5 hours, after which the absorbance was measured at 450 nm using a microplate reader. Cell viability was calculated using the following formula: Cell viability (%) = [A_infection_ – A_bacteria-only_]/[A_cell-control_ – A_blank_] × 100%.

A_infection_: Absorbance of wells containing cells, culture medium, CCK-8 reagent, and bacterial suspension.

A_blank_: Absorbance of wells containing culture medium and CCK-8 reagent.

A_bacteria-only_: Absorbance of wells containing culture medium, bacterial suspension and CCK-8 reagent.

A_cell-control_: Absorbance of wells containing cells, culture medium, and CCK-8 reagent.

### 2.10 Cell adhesion and invasion assay

The cell adhesion and invasion assay were performed according to previously described methods with some modifications (Ogawa et al., 2011). Briefly, human umbilical vein endothelial cells (hUVECs), Immortalized Human Cerebral Microvascular Endothelial Cell Line (hCMEC/D3), Human Trophoblast Cell Line HTR8/svneo (HTR8/svneo) were used in the cell invasion assay. hUVEC and hCMEC/D3 were cultured in Endothelial Cell Medium, supplemented with matched endothelial cell growth supplement, fetal bovine serum (FBS) and penicillin/streptocmycin solution (Sciencell, Carlsbad, CA, United States), HTR8/svneo cells were cultured in RPMI1640 media and supplemented with 10% FBS (Sangon Biotech, Shanghai, China). Cells were incubated at 37 °C in a 5% CO2 atmosphere. GBS internalization by cells was assessed using an antibiotic protection assay. Briefly, cultures were grown in BHI and harvested at the mid-exponential phase of growth by centrifugation for 10 min at 3000×g, washed once by 1×PBS, and resuspended in corresponding cell culture medium. Cells were infected with GBS strains at a multiplicity of infection (MOI) of 1:100 for 2 h. Cells were washed twice with PBS and then incubated with cell culture medium supplemented with lysozyme (1 mg/ml) and penicillin (5 mg/ml) for an additional 2 h. Cells were then washed twice with PBS, lysed using 1% triton-X, and bacterial numbers were counted following inoculation onto BHI agar plates.

The bacterial adhesion assay was performed under the same bacterial inoculum concentration, infection duration and cell culture conditions as the bacterial invasion assay described above. Briefly, sterile coverslips (Φ20 mm) were placed in 12-well plates prior to cell seeding. Target cells were inoculated onto the coverslips and cultured until the cell confluence reached over 90%. Following bacterial infection with the same protocol as the invasion assay, cell monolayers were gently washed five times with PBS to remove non-adherent and loosely adherent bacteria. The coverslips were then fixed with 4% paraformaldehyde, processed with Gram staining, and air-dried at room temperature. Adherent bacteria on the cell surface were observed and imaged under an optical microscope.

### 2.11 *Galleria mellonella* larvae infection model

GBS strains was suspended into BHI and cultured in a shaker for 12 h at 37 °C and 220 rpm. Then strains were reactivated to the logarithmic phase. Bacterial deposits were collected after centrifugation and complete discarding of the supernatant and were adjusted with normal saline to 1×10^8^ CFU/mL. Microsyringes were used to inject different GBS strains in a volume of 10 μL into *Galleria mellonellas* larvae at left posterior proleg. A group of 12 healthy *Galleria mellonellas* were chosen for each strain, larvae were maintained in sterile dishes (6 per dish) and incubated at 37°C. Treated *Galleria mellonellas* were placed in an air incubator at 37 °C and observed at regular intervals for 72 h. Vitality index and mortality rates of infected *Galleria mellonella* larvae at each point were recorded as previously described (Six et al., 2019). In addition, 12 *Galleria mellonella* larvae were assigned to each group. Following infection as described above, hemolymph was collected from the right posterior proleg at multiple time points post-infection before larval mortality occurred. The collected hemolymph was diluted by adding 2µl into 998 µl PBS, and 100µl of the suspension was spread onto the THB agar for colony enumeration. Meanwhile, approximatedly 5µl hemolymph was used to prepare smears for staining and microscopic observation (Banfi et al., 2024). Each test was performed independently in triplicate.

### 2.12 Whole genome sequencing and bioinformatics analysis

Total DNA was extracted using the Rapid Bacterial Genomic DNA Isolation Kit (Sangon biotech, China). Whole genome sequencing was performed using the Illumina HiSeq2500 system (Illumina, San Diego, CA, United States). FastQC software (version 0.11.8) was used for quality control of the pretreated data. SPAdes (version 3.5.0) was used to assemble the sequences (Bankevich et al., 2012). Protein function annotation was performed using the NCBI Blast+ (version 2.2.28) which obtain the functional information from blasting the sequences with the database such as CDD, KOG, COG, NR, NT, PFAM, Swissprot, TrEMBL, CARD, PHI and VFDB ect (Altschul et al., 1997). Given that the maternal vaginal-swab isolate (VC) was the most likely ancestral clone of the infection, it was designated as the reference strain for genomic variant analysis across all isolates. Single nucleotide polymorphism (SNP)/insertion or deletion polymorphism (INDEL) were identified via the HaplotypeCaller of GATK (version 4.1.1.0), while structural variation detection was performed using breseq (version 0.37.0) (McKenna et al., 2010; Deatherage and Barrick, 2014). Then SnpEff (version 4.3T) was employed to annotate mutations based on the annotation data of the reference genome, followed by statistical analysis of the genomic structural distribution of variant sites (Cingolani et al., 2012). FastTree(version 2.1.11) was applied to construct approximately maximum-likelihood phylogenetic trees from aligned nucleotide or amino acid sequences for phylogenetic relationship inference (Vilella et al., 2009). Prophage-associated mobile genetic elements (MGEs) were annotated and characterized using PHASTEST (Wishart et al., 2023). The assembled draft genomes of the isolates were submitted to Database Resources of the National Genomics Data Center, China National Center for Bioinformation.

### 2.13 Transcriptome sequencing and bioinformatics analysis

The GBS strains were first activated in BHI broth overnight at 37°C with shaking at 220rpm. The overnight cultures were subcultured into fresh BHI medium and incubated until the logarithmic growth phase (OD_600_ = 0.5-0.7). Bacterial pellets were collected and used for total RNA extraction via the lysozyme-aided Trizol method, with three biological replicates per group. After extraction, the purity, integrity, and concentration of RNA were determined using a Nanodrop 2000 spectrophotometer, agarose gel electrophoresis, and an Agilent 2100 Bioanalyzer, respectively, to ensure the RNA Integrity Number (RIN) was ≥8.0 and the total RNA amount was ≥1μg. Qualified RNA samples were sent to Sangon Biotech company for sequencing. Bacterial-specific rRNA removal kits were used to eliminate rRNA, followed by cDNA synthesis. After library construction and quality inspection, sequencing was performed on the Illumina MiSeq platform with a sequencing depth of ≥ 6 Gb per sample. Raw sequencing data were evaluated using FastQC and filtered with Trimmomatic (version 0.36) (Bolger et al., 2014). The resulting clean reads were aligned to the reference genome using Bowtie2 (version 2.3.2) (Langmead and Salzberg, 2012). Gene expression levels were quantified by counting reads mapped to gene regions with FeatureCounts (version 1.6.0) and evaluated based on known gene models (Liao et al., 2014). Differential gene expression analysis was conducted using DESeq2 (version1.12.4), raw P-values were adjusted for multiple testing using the Benjamini-Hochberg false discovery rate (FDR) procedure to generate q-values, and the results of this analysis were visualized by volcano plot. Genes were considered significantly differentially expressed when satisfying both criteria: adjusted P-value (q-value) < 0.05 and |log_2_ fold-change| ≥ 1. Subsequently, functional annotation and enrichment analysis were completed by integrating data from the COG, GO, and KEGG databases, along with the ClusterProfiler version 3.0.5.

### 2.14 Real-time fluorescence quantitative PCR

The GBS strains were cultured in BHI broth overnight at 37 °C with shaking. Based on the kit instructions, the total RNA was extracted using a Spin Column bacterial total RNA purification kit (Sangon Biotech, Shanghai, China). Then the cDNA was synthesized from purified total RNA using a PrimeScript RT reagent kit (TaKaRa, Japan). Subsequently, real-time PCRs were performed in a 20 μL system including cDNA, primers, and TB Green premix Ex Taq II (TaKaRa, Japan). The relative expression level of the target gene was calculated using the formula 2^−ΔΔCT^, and *gyrA* was the internal reference gene (Pastuszka et al., 2025). rtPCR validation was performed in three independent biological experiments, with triplicate technical replicates for each sample. **Supplementary Table 1** lists the primer pairs used in this study.

### 2.15 Statistical analysis

Continuous variables were presented as the mean ± standard deviation (SD) or as medians with an interquartile range. Categorical variables were described as frequencies. Normality was assessed for each dataset using the Shapiro-Wilk test. Continuous variables among groups were compared using Student’s t-test or the nonparametric Mann-Whitney U-test. Proportions were compared using a χ-squared test or Fisher’s exact test. For growth curves, differences between strains across time points were analyzed by two-way ANOVA with Bonferroni correction for multiple comparisons. Survival curves were plotted using the Kaplan-Meier method and differences in survival were calculated using the log-rank test. Differences were considered significant at *P < 0.05*. GraphPad Prism 8.0 and R software were used for all statistical analyses.

### 2.16 Ethics statement

This study was approved by the ethics committee of Xinhua Hospital affiliated to Shanghai Jiaotong University school of medicine, Shanghai, People’s Republic of China (XHEC-C-2020-080). This study was conducted using clinical bacterial isolates and retrospectively collected routine clinical data. No additional interventions were performed, and the study did not interfere with patient diagnosis or treatment. All data were de-identified prior to analysis. Accordingly, the requirement for written informed consent was waived by the ethics committee.

## 3 Results

### 3.1 Clinical information

The neonate, male, G3P2, was born at 39 weeks of gestation via vaginal delivery with a birth weight of 3500g. All prenatal examinations of the mother were normal and late-trimester GBS screening was negative. Therefore, no IAP was administered. On the fifth day after birth, the family noticed reduced movement in his right lower limb, and he cried significantly when the right lower limb was touched, but there were no signs of redness, swelling, heat, or ulceration. On the seventh day after birth, the neonate was taken to a local hospital, where a hip ultrasound revealed a large cloudy dark area measuring 12mm×10mm×6mm in the right hip joint (lateral femoral neck), initially suspected to be an abscess. On the 15th day after birth, the patient was transferred to our hospital’s orthopedic department. After admission, joint fluid was aspirated from the joint cavity for culture, and blood samples from both upper limbs were sent for blood culture, both of which showed growth of *S. agalactiae*. On the 19th day after birth, a surgical procedure was performed to clean the lesion in the right hip joint, and a lumbar puncture was performed on the same day to collect cerebrospinal fluid for examination, which indicated purulent meningitis, although the cerebrospinal fluid culture was negative. The patient’s mother is 29 years old, and all prenatal examinations during pregnancy were normal. The patient has been breastfeed since birth. On the eighth day after birth, the mother developed fever (T_max_ 38.9°C), along with swelling and pain in the breast, suspected to be acute mastitis. The microbiology laboratory contacted the patient’s family and collected throat swab samples from the neonate, as well as breast milk and vaginal secretion samples from the mother for culture, all of which isolated *S. agalactiae*. **Figure 1** illustrates the brief clinical course.

**Figure 1:**
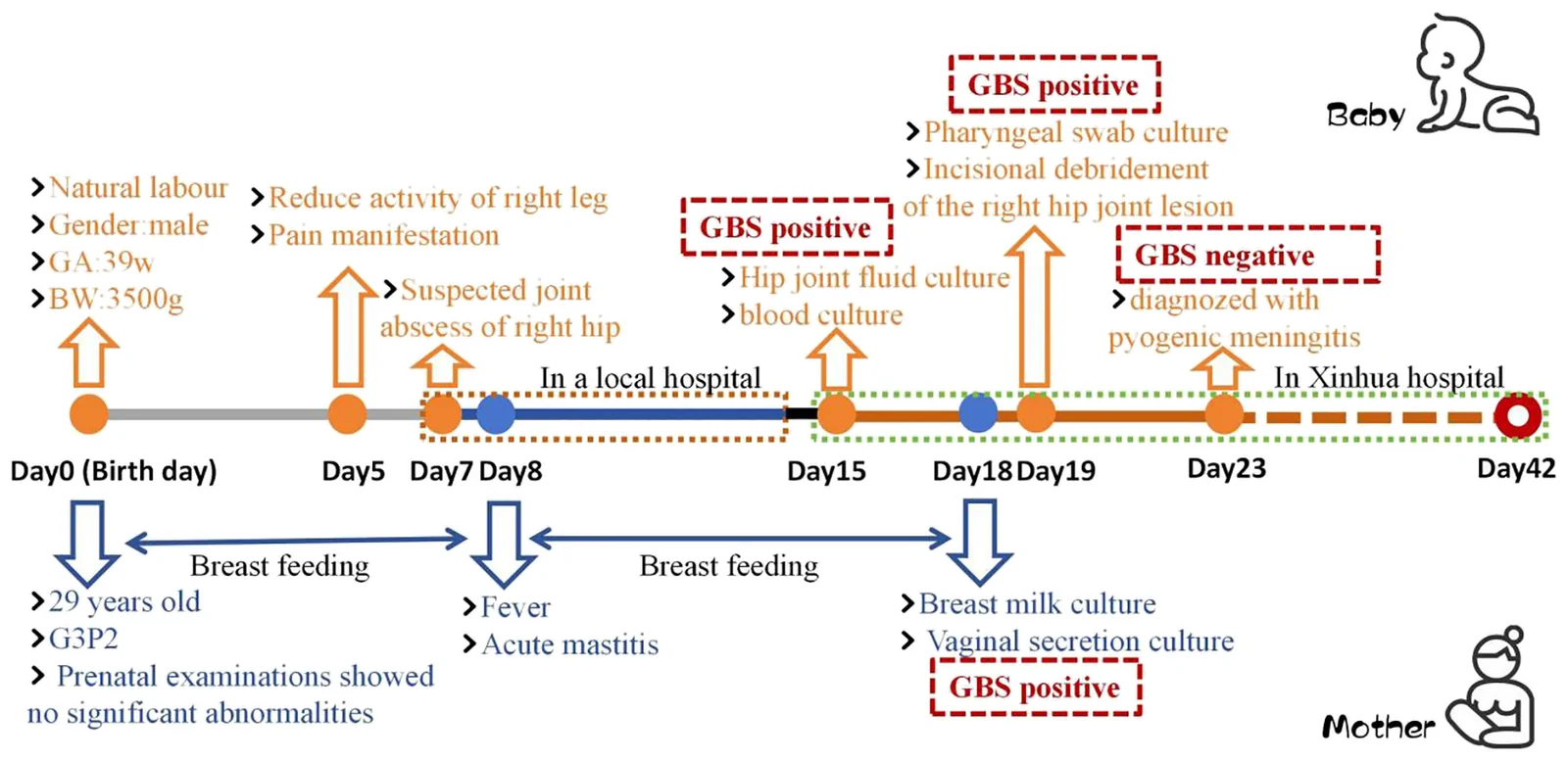
Clinical course of the mother-neonate pair. GA, gestational age; BW, birth weight; GBS, *group B streptococcus*.

### 3.2 The molecular epidemiology characteristics of GBS strains

Serotyping of five GBS strains indicated all were type III. MLST typing revealed that the strains isolated from the joint fluid (SA) and blood (BL) of the neonate belong to a novel sequence type, which subsequently registered and designated as ST2247. While the strains from the neonate’s throat swab (PO), the mother’s vaginal swab (VC), and breast milk (BM) were ST17. All technical replicates of the same isolate yielded completely consistent ST results. ST2247 differs from ST17 at a single locus, the *adhA* allele.

### 3.3 The phenotype characteristics of GBS strains

The drug sensitivity results of the five GBS strains were consistent, all isolates were susceptible to penicillin, ceftriaxone, levofloxacin, linezolid, vancomycin and tigecycline, but resistant to clindamycin, erythromycin and tetracycline. The D test is positive.

Subsequently, two isolates, SA and BM, representing ST2247 and ST17 respectively, were subjected to subsequent experiments. The 24-hour growth curves of ST2247 and ST17 were essentially identical, entering the plateau phase at approximately 10 hours. No significant differences were detected between SA and BM at any individual time point (two-way ANOVA with Bonferroni correction, *P > 0.05* for all comparisons) (**Figure 2A**). The two strains were harvested at the mid-log phase and adjusted to 1 McF. Viable plate counting revealed that the mean bacterial concentrations were (101.1 ± 13.4)× 10^6^ CFU/mL and (103.9 ± 14.0) × 10^6^ CFU/mL, respectively. No statistically significant difference was observed between the two groups (t= -0.43 *P= 0.67*). Serum killing assays indicated baseline inhibition rates at 0 h of 0.32[0.15,2.5] for SA and 1.2[0.16, 1.6] for BM (Mann Whitney U = 4, *P>0.99*). After 1 hour of serum incubation, the inhibition rates reached 1.5[0.5,2.7] for SA and 1.7[1.1,2.3] for BM (Mann Whitney U = 4, *P>0.99*); at 2 hours, the inhibition rates were 2.7[2.0,11.5] and 4.4[4.0-15.9] respectively (Mann Whitney U = 2, *P=0.4*), and at 3 hours, they were 15.6[4.6,30.0] and 17.4[5.6,18.1] respectively (Mann Whitney U = 4, *P>0.99*). None of these differences in overall inhibition rates were statistically significant (**Figure 2B**). The Shapiro-Wilk normality test confirmed that the data of hemolysis assay from both groups followed a normal distribution (W = 0.8995, *P = 0.3839* for group SA; W = 0.7825, *P = 0.0735* for group BM, α = 0.05). So an unpaired two-tailed Student’s t-test was applied. No statistically significant difference was observed in the hemolytic ability of the two strains against rabbit red blood cells (t=0.4641, *P=0.4688*) (**Figure 2C**). When carbon sources were controlled, after 24 hours of cultivation in media containing only GLU or GLU+HA, the number of surviving bacterial strains of ST2247 was higher than that of ST17, though without significant statistical difference (As most groups deviated significantly from normality, the Mann–Whitney U test was used for analysis). Moreover, in medium containing only HA, the number of surviving ST2247 strains 5200[4455,6460] was significantly higher than that of ST17 3880[2080,5100], and this difference was statistically significant (Mann Whitney U = 17.5, *P=0.0424*) (**Figures 2D–F**).

**Figure 2:**
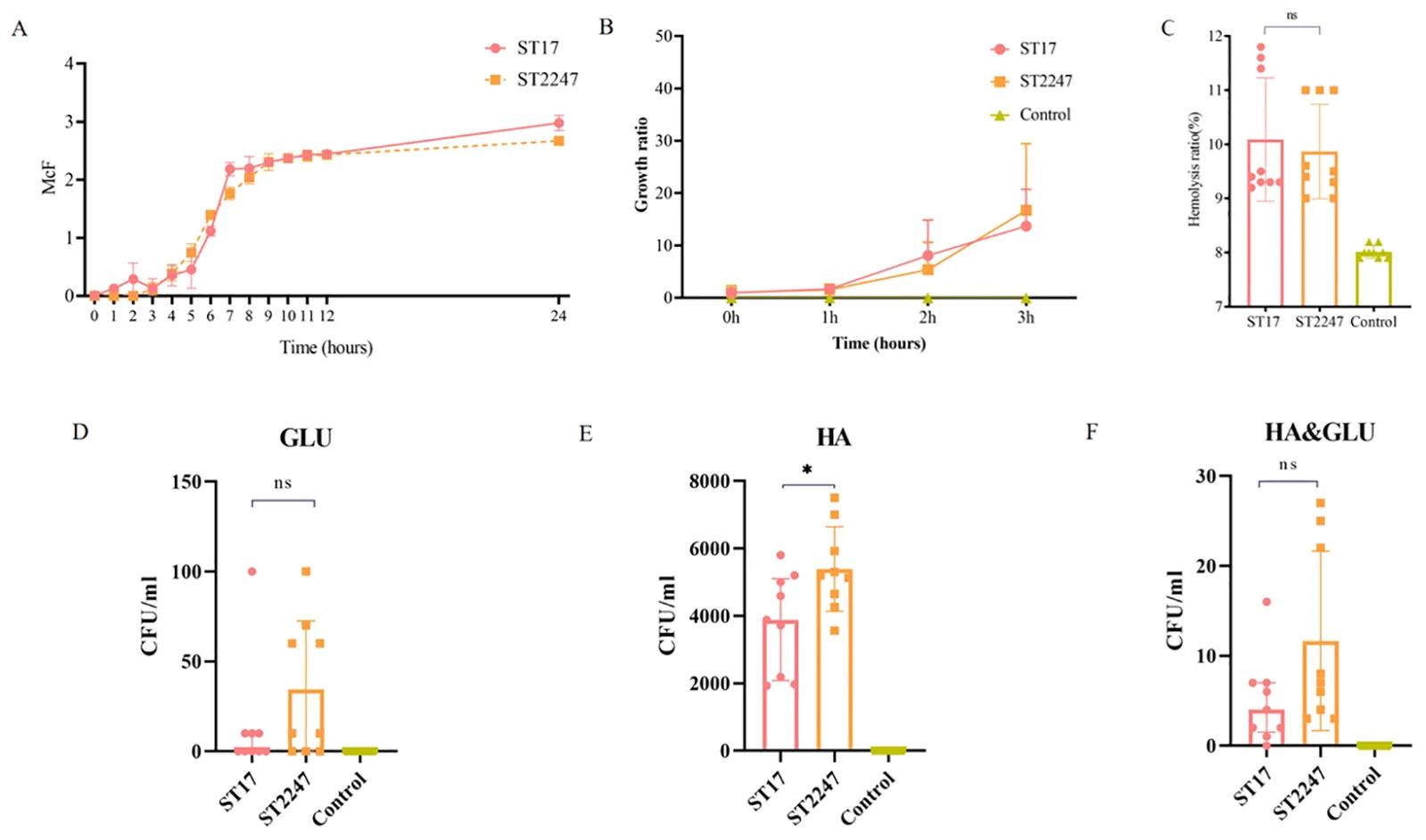
Comparison of growth characteristics between the two strains. **(A)** *In vitro* growth curve of the tested strains cultured in BHI medium, 160rpm, 37°C determined by McFarland turbidity units (McF). The curves overlap due to their nearly identical growth. Data are presented as mean ± SD from three independent biological experiments. For each time point, three technical replicates were measured and averaged within each experiment prior to statistical analysis and plotting. No significant differences were detected between the two strains at any individual time point by two-way ANOVA with Bonferroni correction analysis; **(B)** Kinetics of *in vitro* bactericidal activity against ST17 and ST2247 strains mediated by serum over a 3-hour incubation period. The experiment was independently repeated in three biological batches. Considerable inter-batch variability was observed due to inherent heterogeneity in the composition of serum from different batches, whereas consistent overall trends were detected across all replicates. Data from one representative replicate was presented in the graph. No statistically significant differences in viable bacterial loads were identified between the two groups at 0 h, 1 h, 2 h and 3 h time points (*P > 0.05*); **(C)** Comparison of hemolytic activity against rabbit red blood cells between the two strains. Data are presented as mean ± SD from three independent experiments, each performed in triplicate. Statistical analysis was performed using an unpaired two-tailed Student’s t-test; **(D–F)** Survival under different carbon-source conditions (glucose only, glucose & hyaluronic acid, and hyaluronic acid only). Data are presented as median with interquartile range from three independent experiments, each performed in triplicate. Statistical analysis was performed using the Mann-Whitney U test. *ns, not significant; GLU, glucose; HA, hyaluronic acid.

### 3.4 The virulence of GBS strains

Isolates SA (ST2247) and BM (ST17) were chosen for follow-up assays. The hypersensitive CCK-8 assay showed no statistically significant difference in cytotoxicity between ST17 and ST2247 toward hUVEC, HTR-8/svneo, or hCMEC/D3 cells (**Figures 3A–C**). The Mann-Whitney U test was used to compare intracellular bacterial CFU counts between each groups, as the data were non-normally distributed. For hUVEC, median (interquartile range, IQR) intracellular bacterial CFU was 540 [465, 560] for ST17 strain and 340 [265, 375] for ST2247 strain. Intergroup comparison via two-tailed Mann-Whitney U test produced *P<0.001*, indicating significantly higher intracellular bacterial loads in the ST17 group (**Figure 3D**). For HTR-8/svneo, median intracellular bacterial CFU was 420 [266.5, 474] for ST17 strain and 239 [115, 331] for ST2247 strain (*P=0.0074*), also indicating significantly higher intracellular bacterial loads in ST17(**Figure 3E**). For hCMEC/D3, median intracellular bacterial CFU was 100 [65.5, 145] for ST17 strain and 57[44.5, 82] for ST2247 strain (*P=0.0296*), which also reached statistical significance (**Figure 3F**). Cell climbing assays further revealed that ST17 exhibited superior adhesion capabilities to all tested cell types compared to ST2247 (**Figures 3G–I**).

**Figure 3:**
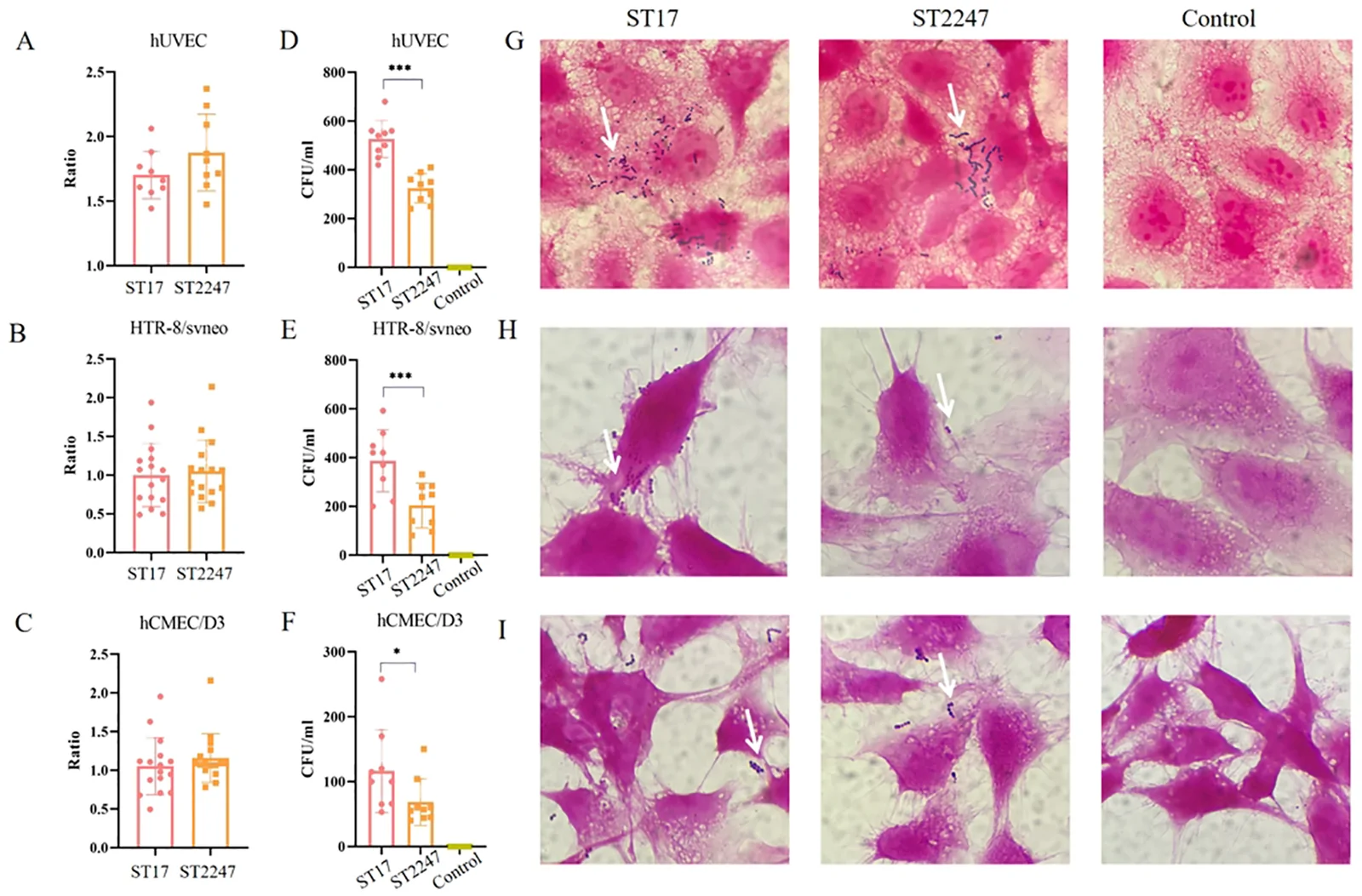
Cytotoxicity, invasive ability, and adhesion of ST17 and ST2247 to various cell lines. **(A–C)**. Cytotoxicity of two GBS strains against hUVEC, HTR-8/svneo, hCMEC/D3 respectively. Data are presented as mean ± SD from three independent experiments, with at least three technical replicates performed in each experiment; **(D–F)**. Invasive ability of two GBS strains against hUVEC, HTR-8/svneo, hCMEC/D3 respectively. Data are presented as median with interquartile range from three independent experiments, each performed in triplicate; **(G–I)**. Adhesion of two GBS strains to hUVEC, HTR-8/svneo, hCMEC/D3 respectively. The Gram-positive coccus indicated by white arrow is *S. agalactiae*. * representing P< 0.05 and *** representing P < 0.001.

The *Galleria mellonella* larvae infection model revealed that ST2247 caused significant vitality reduction in larvae 18 hours post-infection, though no statistically significant difference was observed compared with ST17-infected groups (*P=0.1033*). At 24 (*P=0.0248*) and 48 (*P=0.0433*) hours post-infection, ST2247 induced further decline in larvae vitality, demonstrating statistically significant differences when compared with both ST17-infected and control groups (**Figure 4A**). Survival curve analysis indicated markedly lower survival rates in ST2247-infected *Galleria mellonella* larvae than those infected with ST17 (*P=0.0153*) (**Figure 4B**). Hemolymph was harvested at 2h, 4h, 6h, and 16 h post-infection for dilution plating and colony enumeration, to quantify the GBS burden in *Galleria mellonella* larvae from infection group ST17 and ST2247. The experiment was independently repeated in three biological batches. Considerable inter-batch variability was observed due to inherent heterogeneity, whereas consistent overall trends were observed across all replicates. Data from one representative replicate are presented. At 2 h post-infection, the bacterial load was 12.5[5,41.25] in group ST17 versus 10[5, 43.75] in group ST2247 (Mann-Whitney U = 67.5, *P= 0.81*); at 4 h post-infection, the bacterial load was 15[0, 42.5] in group ST17 and 17.5[0, 40] in group ST2247 (Mann-Whitney U = 65.5, *P= 0.72*); at 6 h post-infection, the bacterial load was 0[0, 22.5] in group ST17 versus 5[0, 10] in group ST2247 (Mann-Whitney U = 67.0, *P=0.77*). At 16 h post-infection, GBS was detectable in only 2 larvae from infection group ST2247, with bacterial loads 230 CFU/μl and 1120 CFU/μl respectively, while GBS loads in the hemolymph of all remaining larvae were below the limit of detection. No statistically significant difference in GBS burden within *Galleria mellonella* larvae was observed between the two groups at any time point (*P<0.05*) (**Supplementary Figure 1A**). However, at 4 hours and thereafter, the proportion of *Galleria mellonella* larvae with bacterial loads above the detection limit (LOD, 5000 CFU/mL) was higher in the ST2247 group than in the ST17 group. At the early stage of infection (2 h post-infection), *Galleria mellonella* larvae infected with ST17 GBS exhibited prominent melanization, whereas this phenotype was less pronounced in the ST2247 group. Conversely, at the mid-stage of infection (16 h post-infection), ST2247 group developed more severe melanization, accompanied by a decrease in larval activity (**Supplementary Figure 2**). On hemolymph smears, it was observed that ST17 infection triggered an increase in inflammatory cells in *Galleria mellonella* larvae at the early stage of infection, with granulocytes seen encapsulating and phagocytosing bacteria. In contrast, no obvious increase in cells was observed during the early stage of infection with the ST2247 strain, and bacteria were scattered throughout the hemolymph (**Supplementary Figure 3**). At 4 and 6 hours post-infection, no significant intergroup differences in hemocyte responses were appreciable by visual inspection alone. Preliminary observations revealed that *Galleria mellonella* larvae had already begun to show a pre-moribund state approximatedly16 hours post-infection in our study. At this point, hemolymph from the control group mainly consisted of granulocytes and plasmatocytes, each accounting for about half of all the hemocytes. Cells were scattered individually and exhibited regular morphology (**Figure 4C**). The overall hemocyte abundance seemed elevated in the ST17 strain infected group compared with the control group (**Figure 4C**). The proportion of granulocytes was moderately higher, with cells exhibiting vacuolation, and mild intercellular aggregation could be dectected. In hemolymph from *Galleria mellonella* larvae infected with ST2247 strain, hemocytes assembled into prominent nodules. Each nodule was dominated by plasmatocytes encircling granulocytes (**Figure 4C**). Gram staining of hemolymph smears from ST2247 infected larvae revealed granulocytes containing engulfed gram positive bacteria; these cells exhibited markedly distinct morphology compared with intact granulocytes (**Figure 4C**).

**Figure 4:**
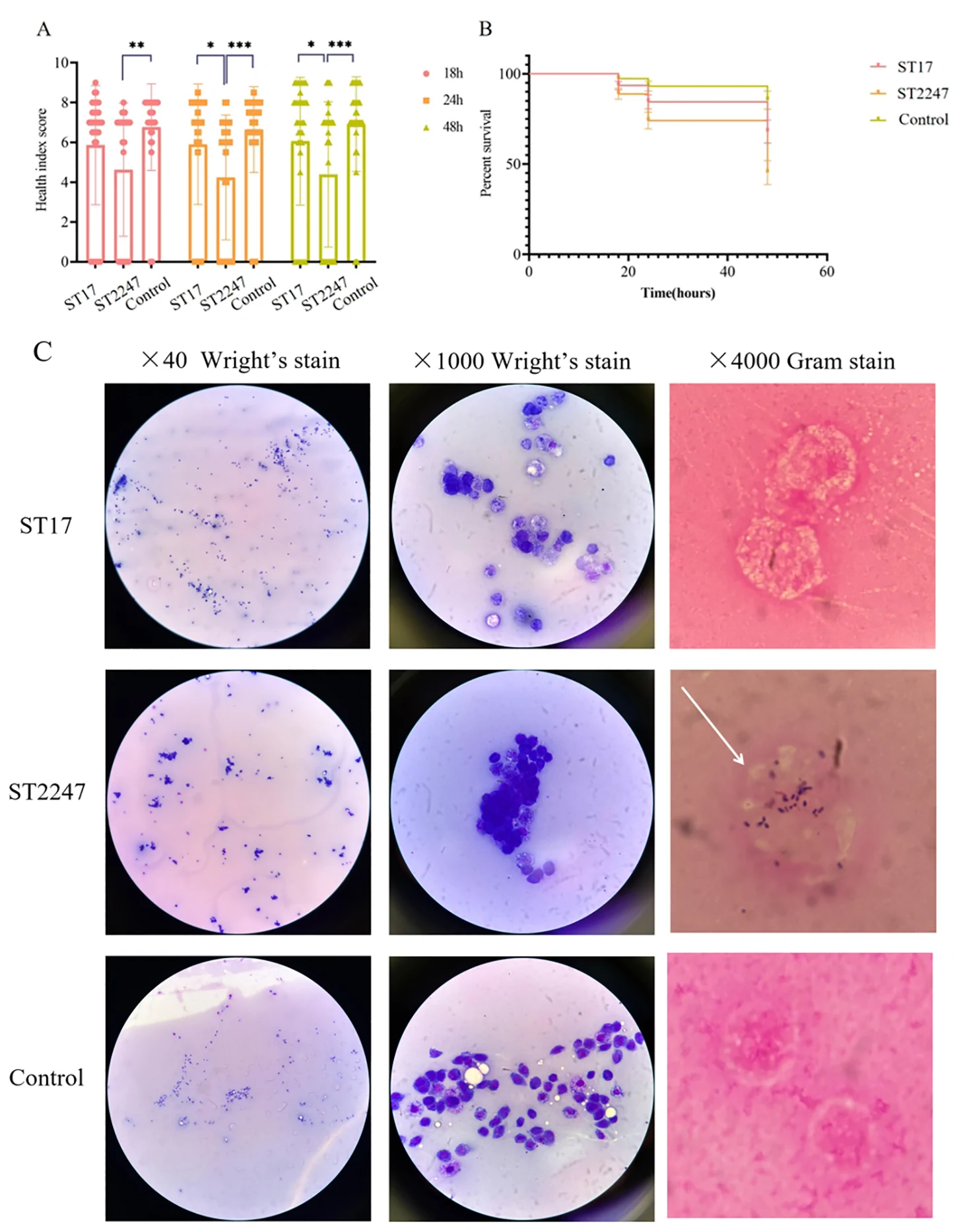
Compare virulence of ST17 and ST2247 by *Galleria mellonella* larvae infection model **(A)** Vitality Index of *Galleria mellonella* larvae infected with ST17 and ST2247 strains at each point. For each group, 12 larvae were used per independent experiment, and three independent biological experiments were performed in total; **(B)** Survival curves of *Galleria mellonella* larvae infected with ST17 and ST2247 strains. For each group, 12 larvae were used per independent experiment, and three independent biological experiments were performed in total; **(C)** Hemolymph smear features of *Galleria mellonella* larvae under different treatments at 16h post infection. At this time point, the total hemocyte count in the infection groups was generally higher than in the control group. However, hemocytes in the ST17 infection group showed mild aggregation, with a relatively high proportion of granulocytes. In contrast, prominent nodulation of hemocytes was observed in the ST2247 infection group, where the hemocyte was dominated by plasmatocytes. On Gram-stained smears, granulocyte from control group retained normal morphology; granulocyte from ST17 infection group showed vacuolated swelling; no obvious bacteria were detected; granulocyte from ST2247 infection group appeared swollen and deformed, with numerous intracellular Gram-positive cocci detectable (indicated by white arrows). * representing P< 0.05, ** representing P < 0.01 and *** representing P < 0.001 respectively.

### 3.5 Whole-genome and transcriptome analysis

Whole-genome sequencing showed all five isolates shared an identical assembled chromosomal size of 2,154,501 bp. The mean sequencing depth ranged from 383.56× to 536.60×, and ≥99.93% of the chromosome was covered at ≥100×, supporting robust identification of genomic variants. SNP and INDEL variant profiling identified three moderate-impact missense mutations in *pepF*, *rpsI* and *adhA*, two synonymous variants in *rapA* and *purR*, alongside two small intergenic indels: one is a single nucleotide insertion (C>CA) situated in the *phoR*-*ptsG* intergenic region, precisely 75bp upstream of the *ptsG* initiation codon, where it likely falls within the *ptsG* promotor, and the other is a single nucleotide deletion (GT>G) located upstream of *rhaR* (**Table 1**). These variants were unique to the invasive isolates BL(ST2247) and SA(ST2247). Using VC(ST17) as reference, phylogenetic tree built from core-genome SNPs divided the isolates into two distinct clades: clade 1 contained BM and PO, while clade 2 contained BL and SA (**Figure 5A**). In silico prediction of antimicrobial resistance genes based on the CARD database demonstrated that all isolates harbored resistance genes *ermB*, *tet(O)*, *mreA*, *APH(3’)- IIIa*, *IsaE*, *mrpF*, *lnuB* and *SAT-4*. Based on VDFB and PHI database, all the five strains exhibited identical virulence profiles. All strains harbored an intact full-length *cyl* operon, complete type III *cps* operon, intact PI-2b pillus island as well as virulence genes such as *lmb*, *scpA*, *hylB*, *cfb*, *srr2* and *hvgA*. All the five isolates harbored an identical intact prophage with a total length of 39.7kb, which was predicted to encode 41 proteins. The prophage shared high similarity with reference phage phiARI0131 (NC_031941). The flanking genomic regions of this prophage consistently contained genes encoding 6-phospho-beta-glucosidase (AscB) and putative transport protein YueF on one side, while IS66-associated proteins were annotated on the opposite flank (**Figure 5B**). Based on clinical data and genomic features, predicted transmission route of the GBS strains in this study were as **Figure 5C** shows.

**Table 1:** Summary of SNPs and INDELs detected in ST2247 strains (reference: VC^*^).

Gene ID	Gene name	Position	Ref	Alt	Type	Effect	HGVS_P
ctg00002_00265	*pepF*	27112	T	G	SNP	missense_variant	p.Ser70Arg
ctg00003_00431	*rapA*	33436	G	A	SNP	synonymous_variant	p.Gly941Gly
ctg00011_01328	*rpsI*	21804	C	T	SNP	missense_variant	p.Ala15Thr
ctg00013_01502	*adhA*	18415	A	G	SNP	missense_variant	p.Val191Ala
ctg00016_01700	*purR*	24581	G	A	SNP	synonymous_variant	p.Ser6Ser
ctg00007_00944-ctg00007_00945	/	24203	T	TTATTA	INDEL	intergenic_region	/
ctg00007_00997-ctg00007_00998	*phoR-ptsG*	68229	C	CA	INDEL	intergenic_region	/
CHR_START-ctg00012_01392	CHR_START-*rhaR*	2056	GT	G	INDEL	intergenic_region	/

*VC is the strain isolated from the mother’s vaginal swab, identified as ST17 by MLST method; Ref, reference; Alt, alternate; HGVS_P, amino acid change.

**Figure 5:**
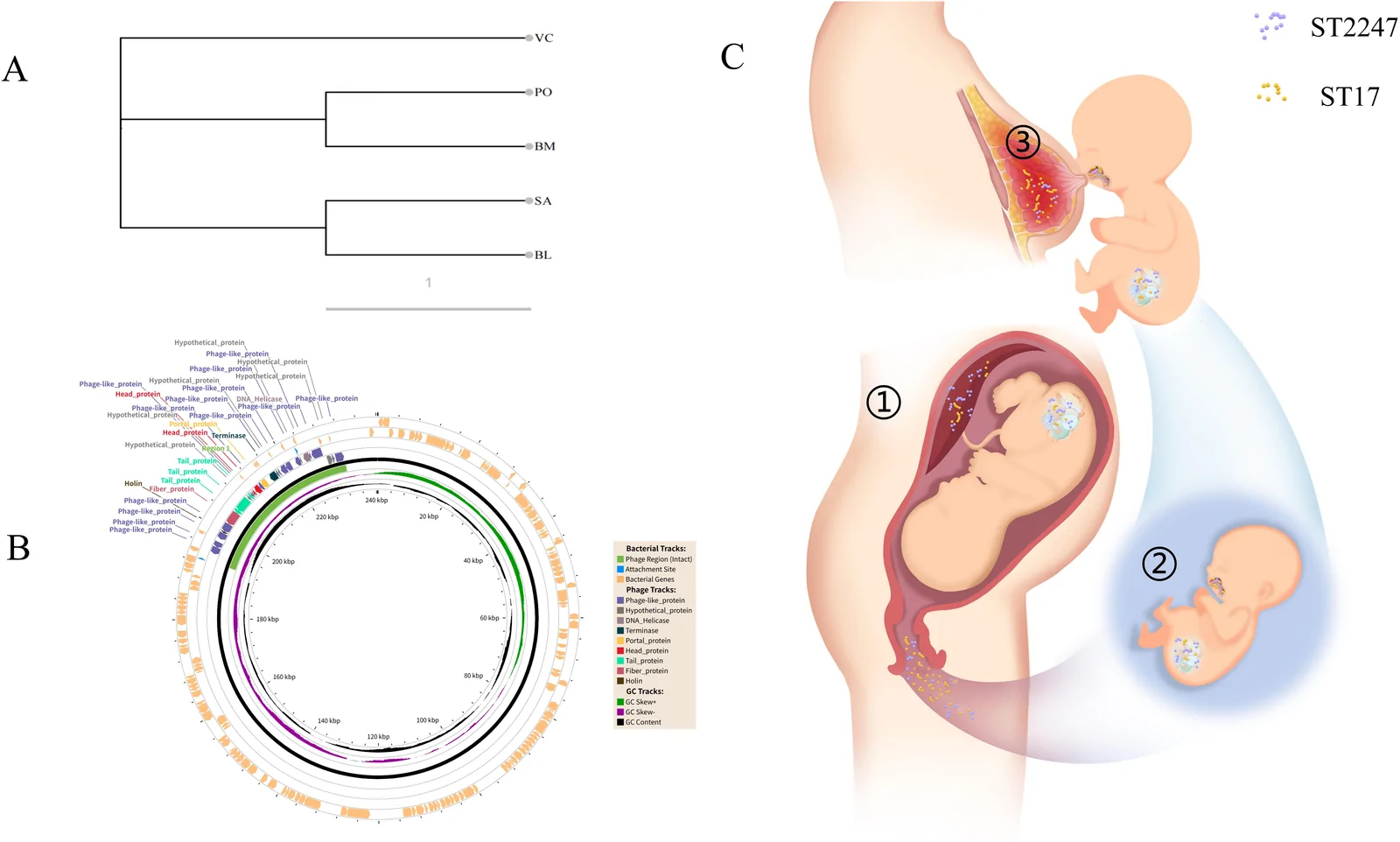
Genomic linkages and likely transmission pathways among five GBS isolates. **(A)** Core SNP-based cladogram of GBS isolates; **(B)** Schematic representation of prophage positions and structures in GBS strains. **(C)** The relationship and the proposed transmission routes of GBS in this case. First, GBS colonizes in the maternal vagina with ST17 as the predominant clone, penetrates the placental barrier and causes fetal infection. Notably, the ST2247 strain becomes the dominant clone causes fetal hip joint infections due to its unique survival capability in the joint cavity environment. Then ST17 GBS predominates in fetal oropharyngeal colonization acquired during vaginal delivery. Finally, The ST17-predominant strain spreads during breast feeding, causing mastitis in the mother. The yellow coccus represent ST17 GBS, the purple coccus represent ST2247 GBS.

Isolates SA (ST2247) and BM (ST17) were chosen for transcriptome analysis. Transcriptome sequencing revealed that ST2247 had 41 upregulated genes and 60 downregulated genes compared to ST17 with raw P (*P<0.05*). Following q-value correction (*q<0.05*) for multiple comparisons, a total of 21 differentially expressed genes (DEGs) were identified. Among them, 13 genes exhibited significantly higher expression levels in ST2247 strain than in ST17 strain, while 8 genes showed significantly lower expression (**Figure 6A**). The differentially expressed genes with |log_2_ fold-change| ≥ 1 were primarily associated with ribosome-encoding genes, ABC transporters, PTS-related genes ect (**Table 2**). Genes *PTSIIBC* and *ElsH* displayed the most significant differential expression between the two groups, with q-values of 2.6×10^-34^, 4.0×10–^12^ and log_2_ fold-changes of 1.95, 1.45, respectively (**Table 2**). The complete DESeq2 differential expression dataset, including log_2_ fold-changes and q values for all detected genes, is provided in **Supplementary Table 2**. The read counts for hyaluronate lyase coding gene *hylB* were zero in both ST17 and ST2247 across all replicates.

**Figure 6:**
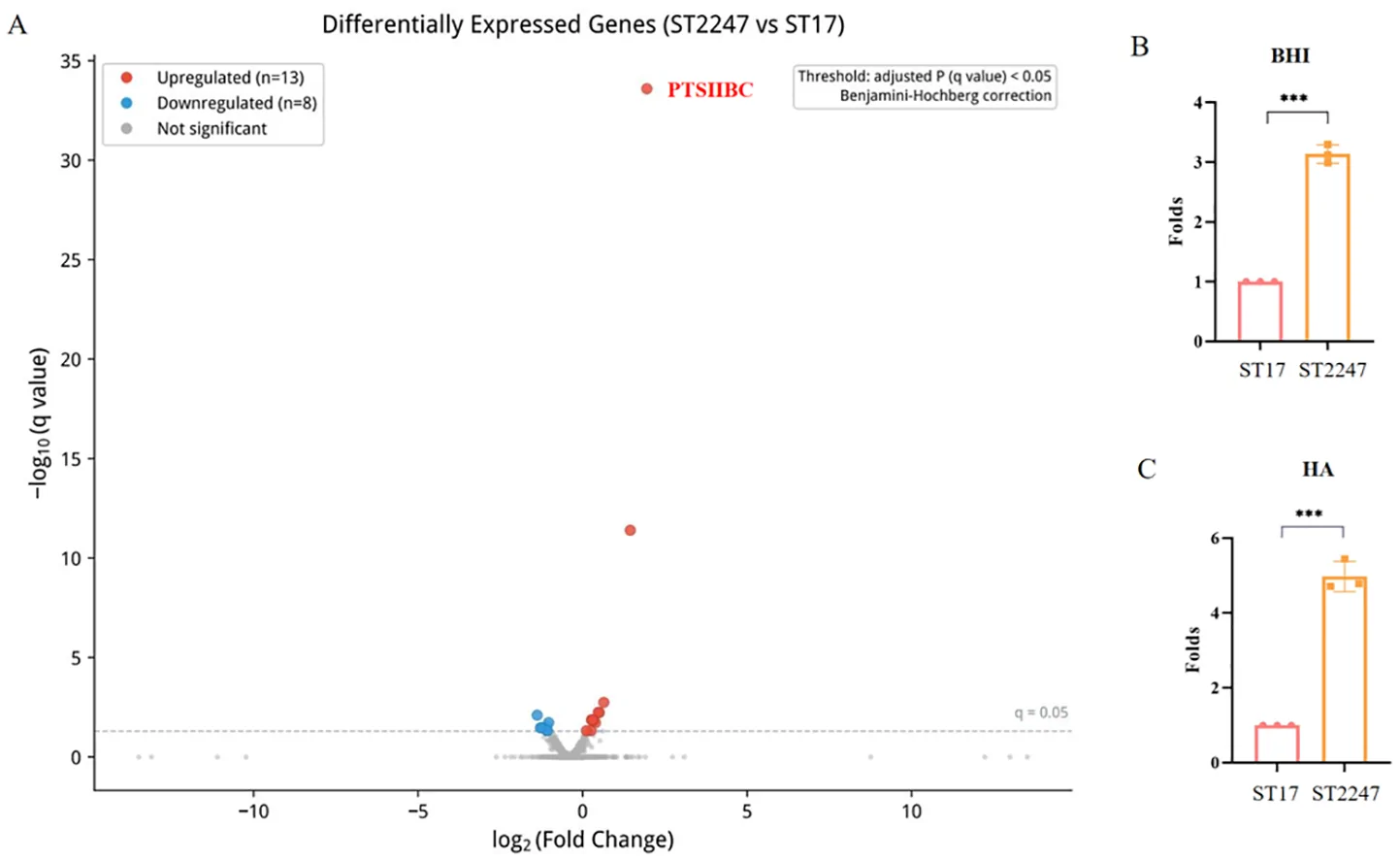
Transcriptome sequencing analysis results of ST17 and ST2247 and *PTSIIBC* gene expression status. **(A)** Volcano plot of differentially expressed genes between GBS ST17 and ST2247 strains. The y-axis represents log_10_(q value), and the x-axis represents log_2_ fold-change. Genes with q value < 0.05 are shown in red (up-regulated) and blue (down-regulated); genes without significant changes are shown in grey; **(B)** Expression levels of *PTSIIBC* genes in ST17 and ST2247 GBS strains cultured under BHI. Data are presented as individual values of three independent biological experiments; each biological experiment was performed with three technical replicates that were averaged prior to analysis; **(C)** Expression levels of *PTSIIBC* genes in ST17 and ST2247 GBS strains cultured under HA treatment. Data are presented as individual values of three independent biological experiments; each biological experiment was performed with three technical replicates that were averaged prior to analysis. *** representing P < 0.001 respectively.

**Table 2:** List of differentially expressed genes between ST2247 and ST17.

Gene id	log_2_ fold-change	pValue	qValue	Result	Gene name	Gene description
AMM49_RS09485	1.95	6.7×10^-37^	2.6×10^-34^	up	*PTSIIBC*	PTS transporter subunit IIBC
AMM49_RS09480	1.45	2.1×10^-14^	4.0×10^-12^	up	*ElsH*	endonuclease/exonuclease/phosphatase family protein
AMM49_RS10205	-1.38	1.2×10^-4^	7.9×10^-3^	down	/	transglycosylase SLT domain-containing protein
AMM49_RS08925	-1.03	6.3×10^-4^	1.9×10^-2^	down	/	glycine betaine/L-proline ABC transporter ATP-binding protein
AMM49_RS03685	-1.09	1.1×10^-3^	3.0×10^-2^	down	/	amino acid ABC transporter ATP-binding protein
AMM49_RS08505	-1.29	1.4×10^-3^	3.4×10^-2^	down	*rpsR*	30S ribosomal protein S18
AMM49_RS06560	-1.25	1.4×10^-3^	3.4×10^-2^	down	*mmuM*	homocysteine S-methyltransferase
AMM49_RS06565	-1.23	1.5×10^-3^	3.4×10^-2^	down	/	amino acid permease
AMM49_RS06885	-1.08	2.3×10^-3^	4.7×10^-2^	down	/	ribosomal-processing cysteine protease Prp
AMM49_RS03790	-1.07	2.6×10^-3^	4.7×10^-2^	down	/	DNA polymerase

The listed genes were defined as differentially expressed with q-value correction and |log_2_ fold-change| ≥ 1.

Subsequently, we validated the expression of genes *PTSIIBC* and *ElsH* using rtPCR. We found that under BHI culture conditions, the expression of the *PTS IIBC* gene in the ST2247 strain was more than three times higher than that in ST17 (**Figure 6B**). More interestingly, in HA solution with concentration at 3mg/ml, the expression of the *PTS IIBC* gene in the ST2247 strain was also approximately five times higher than that in ST17 (**Figure 6C**). However, the expression of *ElsH* did not differ significantly between the two groups. Subsequently, we further validated additional PTS-related genes (including *PTS_mannose* and *PTSIID*) as well as other carbohydrate metabolism-associated genes such as L-lactate dehydrogenase coding gene *L-LDH*, sugar translocase coding gene *gyrA* and hyaluronidase coding gene *hylB*. None of these genes exhibited a statistically significant expression difference between the two groups. Notably, *hylB* transcript was not detected in either strain under the tested conditions (**Supplementary Figure 4**).

## 4 Discussion

In this study, we recovered two GBS strains sharing highly homologous genetic background from a paired mother-neonate case. The isolates from the neonate’s invasive sites (blood and joint fluid), represented a novel sequence type ST2247, which differs from ST17 only at the *adhA* allele. By comparison, isolates from the mother (vagina and breast milk) and oral cavity of the infant belonged to ST17, the well known hypervirulent clone. Previous studies have reported that multiple GBS variants can be isolated from a single anatomical site (Barro et al., 2023), therefore, we also have concerns regarding potential errors or biases in strain identification. To minimize potential errors or bias arising from MLST typing based on single-colony picking, we performed duplicate MLST assays and at least one round of WGS on independently preserved colonies for isolates recovered from each anatomical site. All replicates yielded fully concordant results. Existing epidemiological studies have proposed that GBS typically colonizes the maternal genital tract as a single dominant lineage, which can generate closely related clonal populations through *in vivo* microevolution (Zhou et al., 2022; Pell et al., 2026). Although we cannot exclude the possibility that minor ST variants were present, our results consistently indicate that the identified ST constituted the predominant clone at each anatomical site. *In vitro* experiments showed that the ST17 strains had a stronger ability to invade cell lines, however, in the *Galleria mellonella* larva infection model, the ST2247 strains exhibited greater virulence. Previous studies have shown that bacteria significantly enhance their invasiveness, host adaptability and pathogenicity mainly through mechanisms such as acquiring pathogenicity islands (PAIs), introducing virulent plasmids, or altering regulatory elements that control virulence genes expression (Lee and Song, 2024; Lim et al., 2025; He et al., 2026). However, comparative genomic analysis of all the strains revealed high genome-wide homology. The invasive ST2247 strains lacked acquisition of extra virulence gene islands or operonic rearrangements, with merely five SNPs and three INDELs differentiated it from ST17 strains. Importantly, none of these variants mapped to known virulence genes. Such limited genetic variation seems inadequate to explain their difference in virulence.

The genetic differences identified between ST17 and ST2247 consisted of three moderate-impact missense mutations in *adhA*, *pepF* and *rpsI*, two synonymous variants in *rapA* and *purR*, and two small intergenic indels between intergenic region of *phoR*-*ptsG* and the upstream regulatory region of *rhaR* respectively. The *adhA* is also defines ST2247 as a distinct sequence type. *adhA* encodes an alcohol dehydrogenase implicated in carbohydrate metabolism and central carbon metabolism, which is of potential relevance given the phenotypic differences in carbon source utilization (Zuber et al., 2022). The *pepF* gene in bacteria mainly encodes a highly conserved metal-dependent oligoendopeptidase F (PepF), which functions as a key post-transcriptional regulator of protein metabolism. By specifically degrading intracellular precursor peptides or pheromones, PepF plays a central negative regulatory role in various physiological and metabolic processes and behaviors, including the regulation of antimicrobial peptide synthesis, quorum sensing, competence shut-off, and sporulation (Knoops et al., 2022; Zhang et al., 2024). The *rpsI* gene, which encodes ribosomal small subunit protein S9 (uS9), is a well-characterized target whose mutations mediate bacterial antibiotic resistance, most notably tigecycline resistance. Meanwhile, studies have demonstrated that *purR* mutations affect bacterial virulence (Haeili et al., 2022). A critical determinant of virulence in *purR* mutants is the enhanced production of *SarA*, which limits protease production to promote the accumulation of critical *S. aureus* virulence factors (Alkam et al., 2021). RapA, encoded by *rapA*, is an important transcription cofactor that promotes RNA polymerase (RNAP) recycling by removing core RNAP from DNA, prevents nonspecific RNAP-DNA interactions by allosterically closing the RNAP clamp, and stimulates dissociation of stalled transcription elongation complexes from positively supercoiled DNA, thereby potentially influencing bacterial physiology and metabolism through global transcriptional regulation rather than direct catalysis in specific metabolic pathways (Qayyum et al., 2021; Portman et al., 2022; Inlow et al., 2023). In summary, all these genes are functionally implicated in bacterial fitness. Although the variants identified in our study exhibit a limited magnitude of effect, however, numerous previous studies indicate that SNPs or INDELs located in promoter regions, transcription factor binding sites or small RNA coding regions in bacteria can modulate downstream gene expression and drive phenotypic adaptation by altering transcription factor binding affinity, reshaping cis-regulatory elements, disrupting base pairing between sRNAs and target mRNAs, or modulating transcriptional and translational efficiency (Hua et al., 2022; Fernandez-Fernandez et al., 2026). Minor genetic variations in these non-coding regions are key mechanisms by which bacteria achieve rapid evolution, virulence regulation, and drug resistance under environmental pressure (Barbosa et al., 2021; Brown et al., 2022). Besides, contrary to the long-standing consensus that synonymous variants are “silent” or “neutral”, accumulating evidence shows that they impose pronounced impacts on gene expression, protein function, and bacterial fitness throughout the course of microevolution and adaptive evolution (Bailey et al., 2021; Dhungana et al., 2026). Therefore, although our transcriptome sequencing results did not indicate significant differences in the expression of these genes (except *ptsG*) between the ST17 and ST2247 strain, we cannot exclude the possibility that these genes may still contribute to the changes in fitness and virulence of ST2247 through mechanisms such as affecting protein function via altered enzymatic activity, substrate specificity, or protein stability, as well as through the regulation of upstream or downstream genes. Future studies employing site-directed mutagenesis or complementation assays will be required to determine whether any of these variants contribute to the phenotypic differences observed between the two strains.

The *ptsG* is especially notable in this study because our further transcriptomic analysis results showed that the expression of PTS transporter subunit IIBC encoding gene (*PTSIIBC*) was significantly upregulated in the ST2247 strain. Relative to the ST17 reference isolates, ST2247 isolates harbor an insertion (C>CA) in the upstream region of the *ptsG* gene. As mentioned above, accumulating evidence has established that genetic context, particularly upstream regulatory sequences, exerts a critical influence on gene transcription (Boulas et al., 2023). Therefore, this variant is likely to modulate *ptsG* transcription. Interestingly, rtPCR validation confirmed that under both BHI and hyaluronic acid solution culture conditions, the expression of *PTSIIBC* coding gene was significantly higher in the ST2247 strain than in the ST17 strain.

The glucose-specific enzyme IIBC (EIIBCGlc) encoded by the *ptsG* gene serves as a core component of the PTS system. It primarily mediates the transmembrane transport of glucose and its coupled phosphorylation into glucose-6-phosphate. This protein governs bacterial glucose uptake efficiency, carbon source utilization preference and biological phenotypes, and is associated with bacterial virulence and adaptability (Krahn et al., 2021; Freeman et al., 2025; Taylor et al., 2025). The synovial fluid within the joint cavity is rich in hyaluronic acid, glucose, and other nutrients. However, during the progression of septic arthritis, the rapid proliferation of bacteria leads to the depletion of free monosaccharides such as glucose, accompanied by significant infiltration of leukocytes (Varady et al., 2022; Sacco et al., 2026). This progressively worsening carbon source deprivation, compounded by intensifying immune attack, renders the synovial fluid an unfavorable environment for the survival of GBS during the mid-to-late stages of infection. Previous studies have demonstrated that hyaluronidase encoded by the *hylB* gene degrades hyaluronic acid and is strongly linked to the high invasiveness of GBS (Coleman et al., 2021; Huebner et al., 2022). Nevertheless, transcriptomic and rtPCR data showed undetectable *hylB* transcripts in both ST2247 and ST17 strains, despite the universal carriage of this gene in both strain types. This ruled out differential *hylB* transcription as an explanation for the distinct survival capacity and hypervirulent traits exhibited by ST2247 strains in this study. Therefore, the enhanced survival capacity exhibited by the ST2247 strain in hyaluronic acid solution during phenotypic assays suggests the existence of alternative compensatory mechanisms in this sequence type. Notably, PTS system-related genes, particularly the *PTSIIBC* component, were significantly upregulated at the transcriptional level in the ST2247 strain. Given that the PTS system possesses the dual functions of transporting diverse sugar substrates or sensing and integrating environmental signals (Roth et al., 2024; Banerjee et al., 2026), it is reasonable to infer that these upregulated PTS components may contribute to an important adaptive advantage in the complex nutritional environment of the joint cavity, thereby potentially promoting the overall survival and proliferative capacity of the bacterium. However, the causal relationship between *PTSIIBC* upregulation and this adaptive phenotype has not been functionally verified, and further mechanistic investigation is required.

*In vitro* cellular invasion assays and *Galleria mellonella* larva infection models further confirmed distinct microenvironmental adaptability between the two STs. Although ST17 isolates exhibited greater capacity to invade endothelial cells than ST2247 strains, ST2247 strains displayed higher lethality in the *Galleria mellonella* infection model. Subsequently, we quantified the bacterial loads in the hemolymph of *Galleria mellonella* larvae infected with different strains at multiple time points. Statistical analysis revealed no statistically significant differences in bacterial loads between the two groups at each detected time points. This negative finding may be attributed to the inherent limit of detection of the colony plating assay, which fails to quantify bacterial loads falling below the LOD, and the spatial restriction of micro-sampling from hemolymph, which fails to adequately reflect the overall systemic bacterial burden within the larvae. Further verification could be achieved by refining the experimental approaches, such as constructing strain-specific fluorescent. Interestingly, we found that the temporal dynamics of immune cells infiltration differ markedly induced by the two strains. At the early stage of infection (2 h post-infection), the ST17 strain induced granulocyte and plasmatocyte proliferation in *Galleria mellonella* larvae, while the ST2247 strain did not. Granulocyte phagocytosis was also observed, and the melanization response triggered was significantly stronger than that induced by the ST2247 strain. Studies have demonstrated that the melanization response of *Galleria mellonella* larvae during the early stage of infection efficiently eliminates invading pathogens (Smith et al., 2022). In contrast, ST2247 fails to rapidly trigger robust phagocytosis and melanization in the early infection phase, exhibiting a pronounced delay in immune activation. This lag in immune response may enables ST2247 to evade the host’s early immune clearance, proliferate persistently within the larvae, and ultimately attain a bacterial burden that the host immune system cannot fully eradicate. And, at 16h post-infection, in the group infected with the ST2247 strain, a large number of nodules formed by granulocytes aggregating around plasmatocytes appeared in the hemolymph, and numerous phagocytosed Gram-positive cocci were observed inside the plasmatocytes. Previous studies have demonstrated that nodulation in *Galleria mellonella* larvae is a compensatory immune response elicited when individual phagocytes fail to independently eliminate a high burden of invading pathogens (Garcia-Bustos et al., 2021). This finding indicates that during the middle stages of infection, the immune system of *Galleria mellonella* larvae is overwhelmed and unable to effectively clear the proliferating strain ST2247. These observations may account for the higher mortality observed in *Galleria mellonella* larvae infected with strain ST2247. Furthermore, accumulating evidence indicates that the rapid activation of phagocytes, including neutrophils and plasma cells, during the early phase of human infection plays a critical role in pathogen clearance and clinical prognosis (Diks et al., 2023; Gong et al., 2025). Accordingly, the delayed immune activation phenotype induced by strain ST2247 may also contribute to its capacity to cause bloodstream infection and arthritis in this pediatric patients. Of note, this hypothesis remains to be further validated by functional assays using human immune cells. Notably, all strains isolated in this study harbored identical prophage and virulence gene profiles, indicating that the phenotypic differences observed between strains are unlikely to be attributable to gross genomic differences, such as the acquisition or loss of prophages and virulence-associated genes. Instead, these findings are consistent with the microevolutionary evidence described above and further support the hypothesis that relatively minor regulatory variations may underlie the distinct phenotypic behaviors of the strains. Transcriptome sequencing analyses also revealed that several essential bacterial proteins, such as transglycosylase SLT domain-containing proteins and ABC transporter-associated proteins, which are required for normal bacterial physiology (Leone et al., 2023, Avila-Cobian et al., 2024), were downregulated in ST2247 compared with ST17. Although no direct evidence currently supports that the downregulation of these proteins contributes to bacterial evasion of host immune clearance, this warrants further in-depth investigation.

In terms of infection tracing, the infant developed reduce activity of right leg at 5 days after birth, consistent with the clinical feature of EOD (invasive GBS infection within the first week after birth) (Queensland Health, 2022). Given that vertical mother-to-child transmission constitutes the primary route of EOD transmission and high false-negative rate of GBS culture screening during late pregnancy (El Aila et al., 2010; Matsui et al., 2021; Kumalo et al., 2023; Manuel et al., 2025; Hanan Hamimi et al., 2026), the clinician immediately initiated retrospective sampling for infection tracing from the mother after confirming the infant’s diagnosis of GBS infection. Due to practical clinical limitations, maternal vaginal swab collection was performed about three days subsequent to isolation of the infant’s isolate. Despite this temporal gap in sampling, whole-genome SNP and INDEL analyses revealed that the maternal isolate and the isolates from the infant’s blood and joint fluid shared a high degree of homology, belonging to the same clonal group. As a result all evidence point to that the GBS infection in the infant is highly likely to have originated from maternal-neonatal vertical transmission.

In summary, the ST2247 strain likely represents a microevolutionary derivative of the ST17 clone. The two strain types exhibit a clear divergence in their preference for distinct infection microenvironments. The ST17 strain, which possesses superior colonization ability of the genital tract mucosa, may rapidly induce a host immune reaction, which is associated with its accelerated clearance. However, ST2247 displays delayed induction of the host immune response, which may facilitate its entry into deep invasive sites including the bloodstream and joint fluid. Upon reaching these sites, ST2247 can upregulate its PTS system, making it better adapted to the nutritional and immune pressures encountered there, ultimately allowing it to be selected as the dominant clone in invasive neonatal infections. In addition, the infant was exposed to maternal ST17 strains during vaginal delivery and subsequently colonized by this colonized; breast feeding later transmitted the strain back to the mother’s mammary glands, eventually triggering maternal mastitis (**Figure 5C**).

The core findings of this study were derived from a mother-neonate paired case and the associated GBS isolates. This research model has both unique strengthens and inherent limitations. On one hand, the homologous genetic background of the maternal and neonate’s strains effectively eliminates irrelevant genetic noise, enabling precise identification of minor genetic variants driving virulence divergence and the transcriptomic remodeling triggered by these variants. However, this single-case nature of this study inevitably limits the generalizability of our findings to all ST17 strains or invasive GBS infections. The environmental fitness remodeling of virulence driven by upregulation of key PTS system genes observed in this study awaits validation in a larger cohort of paired isolates encompassing diverse clinical phenotypes, to determine whether this phenomenon represents a widespread pattern of within-host microevolution or merely an isolated event. Specifically, phenotypic variability among isolates within each ST was not comprehensively evaluated, as only one representative strain per ST was included in functional and transcriptomic experiments. Despite high genomic homogeneity within the same ST, intra-ST phenotypic differences may still exist, and thus our conclusions await further validation with a larger collection of isolates.

Overall, based on this naturally-minimal research unit, we systematically characterized the adaptive evolutionary signatures of CC17 GBS throughout colonization, cross-host transmission and invasive pathogenesis, and identified key variant loci potentially associated with neonatal invasive infection, providing molecular evidence to explain why isogenic clonal derivatives produce distinct clinical outcomes. This study also confirms that the hypervirulence of CC17 strains derives not only from their carriage of intrinsic virulence genes, but also from their robust capacity for rapid environmental adaptation, which enabling their persistence within hostile microenvironments. Therefore, pregnant women and infants colonized or infected with CC17 strains require close clinical attention, and specific screening, intervention and treatment protocols should be established for this lineage.

## Data Availability

The datasets presented in this study can be found in online repositories. The names of the repository/repositories and accession number(s) can be found in the article/**Supplementary Material**.
