# Supplementary material for: Adaptive microevolution in CC17 Streptococcus agalactiae from a mother-neonate infection
Source: Front Cell Infect Microbiol. 2026 Sep 14;16:1926326. doi: 10.3389/fcimb.2026.1926326 (PMC13616773; doi:10.3389/fcimb.2026.1926326)
Supplement: Supplementary file 1 [file Table1.docx]

Table S1 Primer Sequences for RT-PCR

| Gene | Forward | Reverse | Product size (bp) |
| --- | --- | --- | --- |
| *hylB* | TCACTTTGCTCTTGCCGCTATG | GCCGTTAGTATTCTTGATGTTGCTAC | 124 |
| *L-LDH* | AAACCAGGTGAAACTCGACTTGAC | GCCGCTACTAAGAAGATACCGTTG | 86 |
| *PTS_mannose* | GCCTTTCTTTGCTCTTGGATTTGC | ACTGATGTAGATGAGGGCGATAGC | 100 |
| *ElsH* | TCATACTCGTCGTGCTCTGGTAG | TCCTCCGCAAATCCTTTCTCAAAC | 102 |
| *gtrA* | ACGCTCCATTCTCTGCAATAGTTG | TGTTTGTAAGCGTTGAAACTGTCC | 116 |
| *PTS* *ⅡD* | TGGTGGCGTTCACAATTCCTTC | CAATGCTGGGATAAGAGCGTAGG | 87 |
| *rnr* | GCAGCCAGGAAGTGAAGTGTTATC | ATTCCGCAGCAAGTCCATAATGTC | 101 |
| *PTS ⅡBC* | TGGTTCCACAGCAAAGCCATCAC | CAGCCTGTAGCAGCAACCCAAG | 150 |
| *gyrA* | TCCTACTGGTGCCTTGGTGATG | CGCTCTTTCCCATTTGAAGTTGTTTC | 120 |

*gyrA* served as the endogenous reference gene

Figure S1 GBS load in *Galleria mellonella* larvae over time. A. Bacterial burden did not differ significantly between larvae infected with ST17 and ST2246 strains at any of the four time points (2h, 4h, 6h, and 16h post-injection). B. The proportion of larvae with detectable GBS (limit of detection: 5×10^3^ CFU/mL) was initially equal at 2 h but became consistently higher in the ST2247-infected group over time. Data shown are from one representative experiment (n=12 per group) of three independent replicates.

Figure S2 Morphological characteristics of *Galleria mellonella* larvae at different time point. In the control group, no obvious color changes were observed in *Galleria mellonella* larvae at different time points (immediately post-injection, 2h post-injection, 16h post-injection) following 1×PBS injection. Melanization occurred at 2 hours post-infection with GBS, and the melanization intensity was greater in larvae infected with the ST17 strain compared with those infected with the ST2247 strain. At 16h post-infection, the melanization response appeared to attenuate in ST17-infected larvae, whereas melanization was further exacerbated in larvae challenged with the ST2247 strain. Each experimental group contained 12 *Galleria mellonella* larvae, and three independent replicates were performed. Since every 6 larvae were placed in one sterile petri dish for incubation, the photographs show 6 of the larvae from one group.

Figure S3 Gram staining of hemolymph smears from *Galleria mellonella* larvae harvested at 2 hours post-injection. A. Massive aggregation of immune cells was observed in larvae infected with ST17 strain (indicated by white arrows). B. Granulocytes were seen aggregating and encapsulating bacterial cells (indicated by black arrows). C. Immune cells were scattered without prominent aggregation following infection with ST2247 strain (indicated by white arrows), with scattered Gram-positive bacteria (indicated by black arrows). Data shown are from one representative experiment of three independent replicates

Figure S4 Comparison of expression levels of the other validated genes (excluding *PTSIIBC*) between ST17 and ST2247 strains. *hylB* expression was not detected (ND) after 40 cycles of amplification, and was assigned a value of zero for graphical presentation. Data are presented as individual values of three independent biological experiments; each biological experiment was performed with three technical replicates that were averaged prior to analysis.
